# STRESSOR VARIETY, SEVERITY, AND IRREGULARITY AND THEIR ASSOCIATIONS WITH DAILY MEMORY LAPSES

**DOI:** 10.1093/geroni/igad104.3115

**Published:** 2023-12-21

**Authors:** Kristin Calfee, Soomi Lee

**Affiliations:** University of South Florida, Tampa, Florida, United States; The Pennsylvania State University, University Park, Pennsylvania, United States

## Abstract

Long-term negative impacts of chronic stress on memory has been well established; however day-to-day associations between stressors and memory have not been fully examined, with lack of understanding on specific manifestations of stressors that may degrade daily memory. This study conceptualized three patterns of daily stressors: variety, severity, and irregularity. Data came from 1,132 adults who participated in an 8-day diary study as part of the Midlife in the United States study. Participants reported 7 types of stressors (e.g., work-related, home-related, discrimination) they encountered, which was summed daily (variety). On days when a stressor was encountered, they reported how stressful it was (severity). Individual standard deviation of stressor severity across the week (irregularity) was also calculated. Memory lapses were reported with 9 items each day. Results from multi-level modeling revealed that, at the within-person level, participants reported more memory lapses on days when they had greater variety (B= 0.126, SE=0.014, p<.001) or higher severity (B=0.071, SE=0.014, p<.001) of stressors. At the between-person level, participants with greater variety (B=0.717, SE=0.052, p<.001), higher severity (B= 0.168, SE= 0.031, p<.001), or greater irregularity (B= 0.141, SE= 0.047, p=.003) of stressors reported more memory lapses on average. These results were found after controlling for sociodemographic and health covariates. These findings highlight that even short-term stress, particularly severe or inconsistent stress may have a negative impact on day-to-day memory. Potential differences between older (age 65+) and younger (below age 65) adults in the relationship between these daily stressors and memory lapses are currently being explored.

